# Prevalence of Self-Reported Hypertension and Antihypertensive Medication Use Among Adults — United States, 2017

**DOI:** 10.15585/mmwr.mm6914a1

**Published:** 2020-04-10

**Authors:** Claudine M. Samanic, Kamil E. Barbour, Yong Liu, Jing Fang, Hua Lu, Linda Schieb, Kurt J. Greenlund

**Affiliations:** ^1^Division of Population Health, National Center for Chronic Disease Prevention and Health Promotion, CDC; ^2^Indiana State Department of Health; ^3^Moffitt Cancer Center, Tampa, Florida; ^4^Division of Heart Disease and Stroke Prevention, National Center for Chronic Disease Prevention and Health Promotion, CDC.

Hypertension, or high blood pressure, is a major risk factor for heart disease and stroke (*1*). The prevalence of hypertension is higher among men than among women, increases with age, is highest among non-Hispanic blacks (blacks) (*2*), and has been consistently highest in the Southeastern region of the United States (*1*). To update prevalence estimates for self-reported hypertension and use of antihypertensive medication, CDC analyzed data from the 2017 Behavioral Risk Factor Surveillance System (BRFSS). The overall (unadjusted) prevalence of self-reported hypertension was 32.4% (95% confidence interval [CI] = 32.1%–32.7%). The age-standardized, median state-specific prevalence of self-reported hypertension was 29.7% (range = 24.3%–38.6%). Overall age-standardized hypertension prevalence was higher among men (32.9%) than among women (27.0%), highest among blacks (40.0%), decreased with increasing levels of education and household income, and was generally highest in the Southeastern and Appalachian states.* Among persons reporting hypertension, the overall unadjusted prevalence of self-reported antihypertensive medication use was 76.0% (95% CI = 75.5%–76.4%). The age-standardized, median state-specific prevalence of antihypertensive medication use among persons with reported hypertension was 59.4% (range = 50.2%–71.2%). Prevalence was higher among women than men, highest among blacks compared with other racial/ethnic groups, and highest among states in the Southeast, Appalachia, and the Dakotas. These findings can help inform CDC’s initiatives to enhance hypertension awareness, treatment, and control across all states.

BRFSS^†^ is an annual, random-digit–dialed telephone survey (both landline and mobile phone), representative of the noninstitutionalized adult population aged ≥18 years of the 50 states, the District of Columbia (DC), and U.S. territories. In 2017, a total of 450,016 adults were interviewed. The present study includes data from the 50 states and DC; the median response rate was 45.9% (range = 30.6%–64.1%).^§^ Respondents were classified as having hypertension if they answered “yes” to the question “Have you ever been told by a doctor, nurse, or other health professional that you have high blood pressure?” Those with borderline and pregnancy-related hypertension were categorized as “no.” Respondents reporting hypertension were classified as currently taking antihypertensive medication if they answered “yes” to the question “Are you currently taking medicine for your high blood pressure?” All analyses incorporated methods to account for the complex survey design. Application of sampling weights accounted for nonresponse, noncoverage, and mobile telephone–only households, and were derived from an iterative proportional weighting (raking) procedure.^¶^

The unadjusted, age-specific, and age-standardized prevalence of self-reported hypertension and antihypertensive medication use were estimated overall, for each of the 50 states and DC, and by sociodemographic characteristics. Prevalence estimates were age-standardized to the 2000 U.S. standard population (*3*). Differences in prevalence across sociodemographic subgroups were tested using chi-squared tests, and differences reported were considered statistically significant for p-values <0.05. All analyses were conducted using SAS-callable SUDAAN (version 11.0.3; RTI International).

During 2017, the overall unadjusted prevalence of hypertension for the 50 states and DC was 32.4% (95% CI = 32.1%–32.7%), representing an estimated 81.7 million adults (Table 1). The age-standardized median state-specific prevalence of hypertension was 29.7% (range = 24.3% [Minnesota] to 38.6% [Alabama and West Virginia]). Age-standardized hypertension prevalences were generally highest in Southeastern and Appalachian states (Figure). Age-specific hypertension prevalence increased with increasing age group (Table 2). The age-standardized prevalence of hypertension was higher among men (32.9%) than among women (27.0%), highest among blacks (40.0%), and decreased with increasing levels of education and household income.

**TABLE 1:** Unadjusted and age-standardized* prevalence of self-reported hypertension (HTN)^†^ and current antihypertensive medication use^§^ among adults aged ≥18 years — Behavioral Risk Factor Surveillance System, 50 U.S. states and the District of Columbia, 2017

Area	Hypertension				Current antihypertensive medication use among adults with hypertension			
	Sample with HTN	Population with HTN (x 1,000)^¶^	% (95% CI)		Sample using antihypertensive medication	Population using antihypertensive medication (x 1,000)^¶^	% (95% CI)	
			Unadjusted	Age-standardized*			Unadjusted	Age-standardized*
**Overall**	**178,312**	**81,674**	**32.4 (32.1–32.7)**	**29.9 (29.6–30.2)**	**146,754**	**61,927**	**76.0 (75.5–76.4)**	**59.6 (58.8–60.3)**
**State**								
Alabama	3,435	1,582	41.9 (40.3–43.4)	38.6 (37.1–40.1)	2,954	1,281	81.1 (79.1–83.1)	70.5 (67.1–73.9)
Alaska	1,245	176	31.8 (29.2–34.5)	31.8 (29.4–34.2)	875	113	64.4 (59.8–69.0)	53.0 (46.7–59.2)
Arizona	6,005	1,655	30.7 (29.8–31.5)	28.0 (27.1–28.8)	4,891	1,236	74.8 (73.2–76.3)	56.0 (53.6–58.4)
Arkansas	2,892	949	41.4 (39.0–43.7)	38.5 (36.1–40.8)	2,547	754	79.6 (76.5–82.8)	69.3 (64.2–74.4)
California	2,854	8,647	28.4 (27.1–29.6)	27.0 (25.9–28.1)	2,060	6,141	71.1 (68.8–73.4)	53.0 (50.0–56.0)
Colorado	3,189	1,130	26.0 (24.9–26.9)	24.8 (23.8–25.7)	2,395	764	69.9 (67.8–72.0)	52.7 (49.6–55.8)
Connecticut	3,991	859	30.5 (29.3–31.6)	27.2 (26.1–28.3)	3,313	658	76.8 (74.8–78.9)	57.3 (54.0–60.6)
Delaware	1,683	263	34.9 (32.9–36.9)	31.1 (29.2–33.0)	1,367	203	77.3 (74.2–80.4)	58.8 (53.5–64.1)
District of Columbia	1,505	149	26.4 (24.8–28.1)	28.2 (26.7–29.6)	1,241	111	74.5 (71.3–77.8)	61.7 (57.3–66.0)
Florida	9,360	5,810	34.6 (33.2–36.0)	29.7 (28.5–31.0)	7,568	4,496	77.5 (75.5–79.5)	58.3 (54.8–61.7)
Georgia	2,520	2,624	33.1 (31.6–34.6)	31.6 (30.2–33.0)	2,153	2,042	77.9 (75.4–80.3)	62.7 (59.0–66.4)
Hawaii	2,657	343	30.6 (29.2–32.0)	28.1 (26.9–29.4)	2,067	257	75.0 (72.5–77.4)	57.9 (54.3–61.5)
Idaho	1,806	379	29.8 (28.1–31.5)	27.5 (26.0–29.0)	1,378	260	69.0 (65.8–72.0)	50.2 (46.2–54.2)
Illinois	2,190	3,187	32.2 (30.8–33.7)	29.9 (28.5–31.3)	1,788	2,410	75.7 (73.3–78.2)	59.8 (55.4–64.1)
Indiana	6,226	1,796	35.2 (34.2–36.3)	32.6 (31.7–33.6)	5,262	1,372	76.5 (74.8–78.2)	60.4 (57.8–63.0)
Iowa	2,906	762	31.5 (30.3–32.6)	28.3 (27.3–29.4)	2,384	589	77.5 (75.5–79.4)	60.7 (57.4–64.0)
Kansas	8,757	718	32.8 (32.0–33.5)	30.5 (29.8–31.2)	7,187	544	75.8 (74.6–77.1)	59.2 (57.3–61.2)
Kentucky	4,214	1,356	39.4 (37.7–41.0)	36.1 (34.6–37.6)	3,600	1,094	80.8 (78.7–82.9)	67.5 (64.1–70.9)
Louisiana	2,208	1,400	39.0 (37.3–40.7)	36.8 (35.2–38.4)	1,849	1,123	80.3 (78.0–82.5)	69.0 (65.3–72.6)
Maine	3,909	376	34.8 (33.4–36.2)	29.9 (28.5–31.3)	3,117	279	74.5 (72(2–76.9)	56.5 (52.7–60.8)
Maryland	5,982	1,522	32.4 (31.2–33.5)	29.8 (28.7–30.9)	5,179	1,211	79.7 (77.8–81.5)	62.6 (59.1–66.1)
Massachusetts	2,475	1,564	28.6 (26.8–30.3)	25.7 (24.3–27.2)	2,053	1,220	78.1 (75.2–81.0)	59.7 (54.4–65.0)
Michigan	4,397	2,697	34.7 (33.6–35.8)	31.3 (30.3–32.3)	3,625	2,067	76.7 (75.0–78.4)	59.4 (56.5–62.2)
Minnesota	5,533	1,134	26.6 (25.8–27.4)	24.3 (23.5–25.0)	4,492	861	76.0 (74.3–77.6)	58.0 (55.3–60.5)
Mississippi	2,621	926	40.8 (38.8–42.7)	38.2 (36.4–40.0)	2,314	750	81.0 (78.3–83.8)	71.2 (66.8–75.5)
Missouri	3,133	1,513	32.0 (30.6–33.4)	29.0 (27.7–30.3)	2,671	1,204	79.7 (77.4–82.0)	64.0 (59.8–68.0)
Montana	2,211	238	29.0 (27.5–30.5)	25.7 (24.2–27.1)	1,750	170	71.8 (68.8–74.7)	51.7 (47.5–56.0)
Nebraska	5,895	443	30.6 (29.5–31.7)	28.2 (27.3–29.2)	4,957	348	78.6 (76.8–80.4)	61.5 (58.3–64.7)
Nevada	1,471	757	32.6 (30.5–34.8)	30.0 (28.1–32.0)	1,149	548	72.5 (68.9–76.2)	55.1 (49.2–61.1)
New Hampshire	2,284	324	30.0 (28.4–31.6)	25.9 (24.4–27.4)	1,915	257	79.7 (77.0–82.3)	62.2 (56.0–68.3)
New Jersey	4,897	2,305	33.0 (31.6–34.4)	30.1 (28.8–31.4)	4,096	1,750	76.0 (73.7–78.4)	58.3 (54.7–62.0)
New Mexico	2,496	484	30.5 (29.0–32.0)	28.0 (26.6–29.4)	1,952	353	73.2 (70.5–75.8)	57.1 (52.9–61.3)
New York	4,329	4,574	29.4 (28.3–30.5)	27.1 (26.2–28.1)	3,485	3,449	75.6 (73.7–77.5)	57.4 (54.6–60.2)
North Carolina	2,002	2,775	34.7 (33.0–36.5)	31.8 (30.2–33.3)	1,662	2,217	80.0 (77.6–82.5)	64.1 (59.9–68.4)
North Dakota	2,813	173	29.5 (28.2–30.8)	28.2 (27.0–29.4)	2,401	135	78.2 (75.9–80.6)	63.2 (59.2–67.3)
Ohio	5,394	3,130	34.7 (33.5–35.9)	31.4 (30.2–32.6)	4,618	2,433	77.9 (75.9–79.9)	61.5 (58.3–64.6)
Oklahoma	3,176	1,124	37.7 (36.2–39.2)	35.4 (34.0–36.7)	2,719	874	77.8 (75.6–80.0)	64.0 (60.6–67.5)
Oregon	1,835	987	30.1 (28.7–31.5)	27.2 (25.9–28.5)	1,374	699	71.0 (68.4–73.5)	53.3 (49.5–57.0)
Pennsylvania	2,337	3,295	32.6 (31.1–34.1)	28.9 (27.6–30.2)	1,896	2,586	78.6 (76.4–80.9)	60.9 (56.9–64.8)
Rhode Island	2,303	280	33.1 (31.4–34.8)	29.9 (28.3–31.5)	1,969	226	81.0 (78.4–83.7)	65.5 (60.3–70.6)
South Carolina	5,632	1,498	38.1 (36.9–39.3)	34.4 (33.3–35.6)	4,916	1,206	80.6 (78.9–82.4)	68.5 (65.2–71.8)
South Dakota	2,862	203	30.8 (28.9–32.7)	28.0 (26.2–29.7)	2,420	161	79.4 (76.3–82.5)	64.8 (59.0–70.5)
Tennessee	2,638	2,012	38.7 (36.9–40.4)	35.5 (33.9–37.2)	2,210	1,580	78.6 (76.0–81.1)	65.0 (60.9–69.1)
Texas	5,299	6,853	32.5 (30.8–34.2)	31.9 (30.3–33.5)	4,446	4,958	72.4 (69.4–75.3)	57.5 (53.5–61.6)
Utah	3,044	534	24.5 (23.4–25.5)	25.4 (24.5–26.4)	2,224	359	67.4 (65.1–69.7)	52.3 (49.6–55.1)
Vermont	2,313	153	30.4 (28.9–31.9)	26.4 (25.1–27.8)	1,804	112	73.5 (71.0–76.1)	51.7 (47.8–55.6)
Virginia	3,895	2,136	32.4 (31.1–33.6)	30.3 (29.1–31.5)	3,245	1,613	75.7 (73.5–77.9)	58.3 (55.1–61.5)
Washington	4,840	1,700	29.5 (28.6–30.5)	27.6 (26.6–28.5)	3,696	1,184	69.9 (68.0–71.7)	54.5 (51.9–57.2)
West Virginia	2,769	631	43.5 (28.6–30.5)	38.6 (37.0–40.2)	2,380	502	79.6 (77.5–81.7)	61.7 (58.4–65.1)
Wisconsin	2,143	1,387	30.8 (29.2–32.4)	27.9 (26.4–29.4)	1,743	1,041	75.4 (72.6–78.2)	57.0 (52.4–61.5)
Wyoming	1,741	138	30.8 (29.2–32.4)	28.5 (27.0–30.0)	1,397	98	71.7 (68.8–74.7)	53.5 (49.4–57.6)
Median	—	—	32.2	29.7	—	—	76.7	59.4
Range	—	—	24.5–43.5	24.3–38.6	—	—	64.4–81.1	50.2–71.2

**Abbreviation**: CI = confidence interval.

* Age standardized to the 2000 U.S. projected population using three age groups: 18–44, 45–64, and ≥65 years.

^†^ Hypertension was defined as an affirmative response to “Have you ever been told by a doctor, nurse, or other health professional that you have high blood pressure?” Preeclampsia or borderline high or prehypertensive was categorized as “no.”

^§^ Current antihypertensive medication use was defined as affirmative response to “Are you currently taking medicine prescribed by a doctor or other health professional for your high blood pressure?”

^¶^ Weighted number of adults in the population with hypertension or currently using antihypertensive medication.

**FIGURE:**
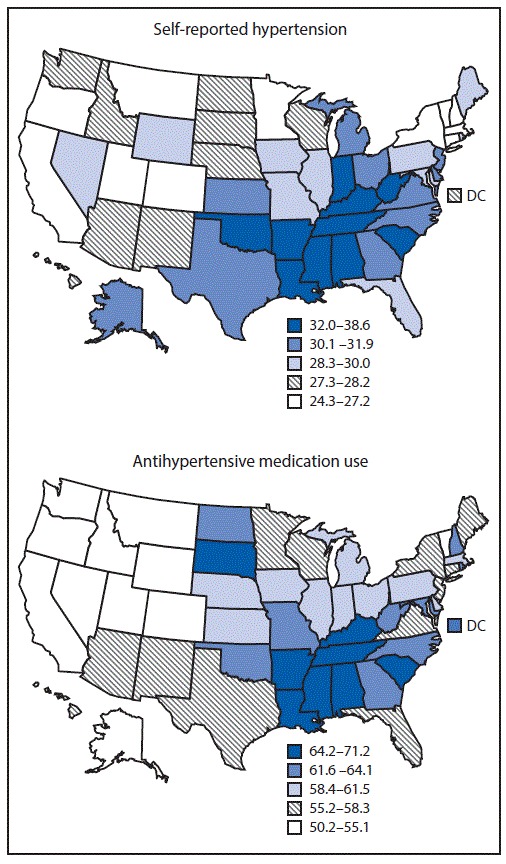
Age-standardized percentage of self-reported hypertension and antihypertensive medication use among adults aged ≥18 years, by state — Behavioral Risk Factor Surveillance System, United States, 2017 **Abbreviation:** DC = District of Columbia.

**TABLE 2:** Unadjusted and age-standardized* prevalence of self-reported hypertension (HTN)^†^ and antihypertensive medication use^§^ among adults aged ≥18 years, by selected characteristics — Behavioral Risk Factor Surveillance System, United States, 2017

Characteristic	Hypertension				Antihypertensive medication use among adults with hypertension			
	Sample with HTN	Population with HTN (x 1000)^¶^	% (95% CI)		Sample using antihypertensive medication	Population using antihypertensive medication (x 1,000)^¶^	% (95% CI)	
			Unadjusted	Age-standardized*			Unadjusted	Age-standardized*
**Overall**	**178,312**	**81,674**	**32.4 (32.1–32.7)**	**29.9 (29.6–30.2)**	**146,754**	**61,927**	**76.0 (75.5–76.4)**	**59.6 (58.8–60.3)**
**Age group (yrs)**								
18–44	18,432	16,429	14.1 (13.7–14.5)	14.1 (13.8–14.5)	7,512	6,195	37.9 (36.5–39.2)	37.9 (36.5–39.2)
45–64	66,699	34,048	40.5 (40.0–41.0)	40.5 (40.0–41.0)	53,783	27,085	79.6 (78.9–80.3)	79.6 (78.9–80.3)
≥65	93,181	31,198	60.5 (60.0–61.1)	60.5 (60.0–61.1)	85,459	28,647	92.0 (91.5–92.4)	92.0 (91.5–92.4)
Sex*								
Men	81,648	42,260	34.5 (34.0–34.9)	32.9 (32.5–33.3)	64,010	30,136	71.5 (70.7–72.2)	56.7 (55.8–57.6)
Women	96,569	39,363	30.4 (30.0–30.8)	27.0 (26.6–27.3)	82,669	31,747	80.8 (80.1–81.4)	64.0 (62.7–65.2)
Race/Ethnicity*								
White, non-Hispanic	136,668	53,179	34.0 (33.7–34.3)	29.0 (28.7–29.3)	113,525	41,278	77.7 (77.2–78.2)	59.0 (58.1–59.9)
Black, non-Hispanic	18,628	12,127	41.1 (40.1–42.1)	40.0 (39.2–40.9)	16,116	9,649	79.6 (78.3–80.9)	68.1 (66.2–70.0)
Hispanic	9,081	9,510	23.9 (23.0–24.7)	28.2 (27.3–29.1)	6,359	6,133	64.8 (62.8–66.8)	54.0 (51.9–56.0)
American Indian/Alaska Native, non-Hispanic	3,624	976	38.8 (36.4–41.3)	37.1 (34.7–39.5)	2,784	690	70.7 (66.7–74.7)	58.6 (53.6–63.5)
Asian, non-Hispanic	2,290	2,659	19.6 (17.8–21.4)	23.8 (21.9–25.8)	1,786	1,835	69.2 (64.5–73.9)	58.0 (52.8–63.0)
Native Hawaiian/Pacific Islander, non-Hispanic	316	127	26.4 (21.2–31.7)	33.0 (28.3–38.0)	200	87	68.4 (59.1–77.6)	54.9 (45.8–63.6)
Multiracial, non-Hispanic	3,373	1,060	30.1 (28.3–32.0)	31.6 (29.9–33.4)	2,504	731	69.1 (65.9–72.3)	56.7 (52.8–60.6)
Other, non-Hispanic	880	368	33.1 (28.8–37.3)	28.9 (25.3–32.8)	703	276	75.2 (67.9–82.5)	54.9 (45.4–64.0)
Education level*								
Less than high school	15,316	13,232	39.1 (38.1–40.2)	35.4 (34.4–36.3)	12,605	10,020	75.9 (74.4–77.4)	58.6 (56.4–60.8)
High school or equivalent	54,498	24,742	35.2 (34.6–35.7)	32.3 (31.8–32.8)	45,423	18,944	76.7 (75.9–77.6)	59.6 (58.4–60.9)
More than high school	107,886	43,411	29.5 (29.2–29.9)	27.5 (27.2–27.8)	88,234	32,756	75.6 (74.9–76.2)	59.8 (58.8–60.8)
Household income*								
<$15,000	17,836	9,145	40.7 (39.6–41.8)	37.9 (36.9–39.0)	14,384	6,889	75.5 (73.9–77.1)	61.5 (59.3–63.7)
$15,000 to <$25,000	28,614	13,017	36.9 (36.1–37.7)	34.3 (33.6–35.1)	23,605	9,895	76.1 (74.9–77.4)	59.7 (57.9–61.5)
$25,000 to <$35,000	17,502	7,731	35.5 (34.5–36.6)	31.9 (30.9–32.9)	14,589	5,928	76.8 (75.3–78.4)	60.4 (57.5–63.2)
$35,000 to <$50,000	22,129	9,213	33.1 (32.3–34.0)	29.9 (29.1–30.7)	18,451	7,029	76.4 (75.0–77.8)	56.9 (54.9–58.8)
≥$50,000	61,667	29,012	28.2 (27.8–28.7)	26.9 (26.5–27.3)	49,890	21,529	74.3 (73.5–75.1)	59.7 (58.5–60.9)

**Abbreviation**: CI = confidence interval.

*Age standardized to the 2000 U.S. projected population using three age groups: 18–44, 45–64, and ≥65 years.

^†^ Hypertension was defined as an affirmative response to “Have you ever been told by a doctor, nurse, or other health professional that you have high blood pressure?” Preeclampsia or borderline high or pre-hypertensive was categorized as “no.”

^§^ Current antihypertensive medication use was defined as affirmative response to “Are you currently taking medicine prescribed by a doctor or other health professional for your high blood pressure?”

^¶^ Weighted number of adults in the population with hypertension or currently using antihypertensive medication.

Among those reporting hypertension, the overall, unadjusted prevalence of antihypertensive medication use was 76.0% (95% CI = 75.5%–76.4%), representing an estimated 61.9 million adults (Table 1). The age-standardized, median, state-specific prevalence of antihypertensive medication use was 59.4% (range = 50.2% [Idaho] to 71.2% [Mississippi]). Age-standardized prevalence of antihypertensive medication use was highest in the Southeastern and Appalachian states, as well as the Dakotas (Figure). The age-specific prevalence of antihypertensive medication use also increased with increasing age (Table 2), was highest among blacks (68.1%), was higher among women (64.0%) than among men (56.7%), and did not vary by education or household income level.

## Discussion

During 2017, approximately one third (82 million) of U.S. adults reported having hypertension, and an estimated three quarters of those with hypertension (62 million) reported using antihypertensive medication. Age-standardized prevalence of hypertension varied widely by state, remaining highest in the Southeast and among men and blacks. Age-standardized prevalence of antihypertensive medication use also increased with increasing age, was highest among blacks, and was higher among women than among men.

The overall age-standardized self-reported hypertension prevalence of 29.9% was similar to that reported based on 2011–2015 BRFSS data (29.8%) (*1*) and measured hypertension prevalence of 29% based on data from the 2015–2016 National Health and Nutrition Examination Survey (*2*). Also consistent with other reports, hypertension prevalence decreased with increasing income (*4*) and education level (*1*) and was highest in Southeastern and Appalachian states (*1*,*2*). The overall, age-standardized prevalence of antihypertensive medication use (59.6%) was also similar to estimates from the 2011–2015 BRFSS, ranging from 63.0% in 2011 to 61.8% in 2015 (*1*). Like hypertension prevalence, medication use prevalence was highest in Southeastern and Appalachian states. In the present study, prevalence of medication use was also highest in the Dakotas, despite a midrange prevalence of hypertension in these states. Prevalence of antihypertensive medication use was higher in older age groups, highest among blacks, and higher among women than men. This overall gender difference has been reported previously (*1*), but the reasons are unclear. Data from Medicare Part D beneficiaries aged ≥65 years suggest that antihypertensive medication nonadherence is similar for men (25.8%) and women (26.7%) (*5*). More information is needed to examine the relationship between the prevalence of self-reported hypertension and that of antihypertensive medication use.

The findings in this report are subject to at least three limitations. First, data were self-reported. The lack of documented diagnosis of hypertension based on historic blood pressure measurements does not allow for precise assessment of hypertension; however, the results were similar to data from previous reports based on both self-report (*1*) and measured hypertension (*2*). Second, low median response rates across states might limit the representativeness of the 2017 BRFSS sample and potentially result in either under- or overestimates of prevalence, although application of sampling weights is likely to reduce some nonresponse bias. Finally, findings are representative of noninstitutionalized civilian persons only and would exclude those living in nursing homes, prisons, and other institutions.

This report provides the most recent state-level surveillance data on prevalence of self-reported hypertension and antihypertensive medication use among persons reporting hypertension. Hypertension prevention and control is a priority of CDC’s state and local funding for heart disease and stroke prevention** and one of the important elements of the Million Hearts initiative (*6*). CDC has been working closely with states to enhance hypertension management through a strategy of team-based care in which two or more health care providers work collaboratively with each patient. These teams may include doctors, nurses, pharmacists, dietitians, community health workers, and other health care providers. This approach is often multidisciplinary with a team working to educate patients, identify risk factors, provide treatments, and sustain ongoing conversations with patients. This strategy can result in multiple opportunities for intervention for better blood pressure control (*7*),^††^ with the ultimate goal of reducing disparities in hypertension awareness, treatment, and control across the United States.

**Summary**

**What is already known about this topic?**

Prevalence of hypertension increases with increasing age and is higher among men than women and among non-Hispanic blacks than among other racial/ethnic groups; prevalence has been consistently higher in the Southeastern and Appalachian regions of the United States.

**What is added by this report?**

Analysis of 2017 Behavioral Risk Factor Surveillance System data found that approximately one third of U.S. adults reported having hypertension, and an estimated 75% of those reporting having hypertension reported using antihypertensive medication. The prevalence of these factors varied widely by state and was generally highest in the Southeastern and Appalachian states.

**What are the implications for public health practice?**

A multidisciplinary team-based strategy working to educate patients, maintain dialogue over time, and identify risk factors can provide intervention opportunities for better blood pressure control and could reduce disparities in hypertension awareness, treatment, and control across the United States.

## Footnotes

* https://www.arc.gov/appalachian_region/TheAppalachianRegion.asp.

† https://www.cdc.gov/brfss/index.html.

§ https://www.cdc.gov/brfss/annual_data/2017/pdf/2017-sdqr-508.pdf.

¶ https://www.cdc.gov/brfss/annual_data/2017/pdf/weighting-2017-508.pdf.

** https://www.cdc.gov/dhdsp/programs/index.htm.

†† https://www.cdc.gov/dhdsp/pubs/guides/best-practices/team-based-care.htm.
